# Acquired Digital Fibrokeratoma

**DOI:** 10.7759/cureus.47997

**Published:** 2023-10-30

**Authors:** Oscar V Navea, Maria B Navea, Raul De la Fuente

**Affiliations:** 1 General Practice, Universidad de los Andes, Santiago, CHL; 2 General Practice, Universidad de Chile, Santiago, CHL; 3 Dermatology, Hospital Clínico de la Universidad de Chile, Santiago, CHL

**Keywords:** occupational diseases, occupational pathology, cutaneous horn, acquired fibrokeratoma, acquired digital fibrokeratoma

## Abstract

Acquired digital fibrokeratoma is a rare, benign tumor that mostly occurs on the fingers and toes and may appear to be a supernumerary rudimentary digit. It generally affects adult men and appears as a dome-shaped papule although it can also be elongated or pedunculated. Trauma is believed to be a triggering factor in some cases. We report a male patient with an acquired digital fibrokeratoma on a finger, shaped like a cutaneous horn, and a history of minimal repeated trauma and spontaneous remissions not previously described in the literature.

## Introduction

Acquired digital fibrokeratoma (ADFK) is a benign tumor that occurs on the fingers or toes and occasionally on the palms and soles, usually in adults [1].

Clinically, ADFK typically presents as a small, single, skin-colored papule on the finger or toe. It is generally surrounded by a collar of slightly raised skin called the “fossa”, and is usually asymptomatic [2].

Histopathologically, ADFK shows an epithelium with marked orthokeratosis and acanthosis. The network of interpapillary ridges appears thickened and generally branched. The enveloped dermis is composed of a thick bundle of interwoven collagen, which is oriented parallel to the longitudinal axis of the lesion. Elastic fibers are thin and few, if not completely absent, and the tumor tends to be highly vascularized. Curative treatment consists of complete surgical excision [2].

## Case presentation

A 47-year-old male patient, a welder by trade, presented approximately 15 years ago with a cutaneous tumor lesion on the lateral aspect of the second phalanx of the right middle finger. The lesion began as a firm, asymptomatic papule, which the patient initially traumatized repeatedly with the intention of removing it. The tumor grew and, although asymptomatic, it bothered him at work, for which it was necessary to wear gloves. He reports that this lesion has presented multiple subtotal spontaneous remissions, mainly in winter, leaving a papule from which the tumor re-emerges.

**Figure 1:**
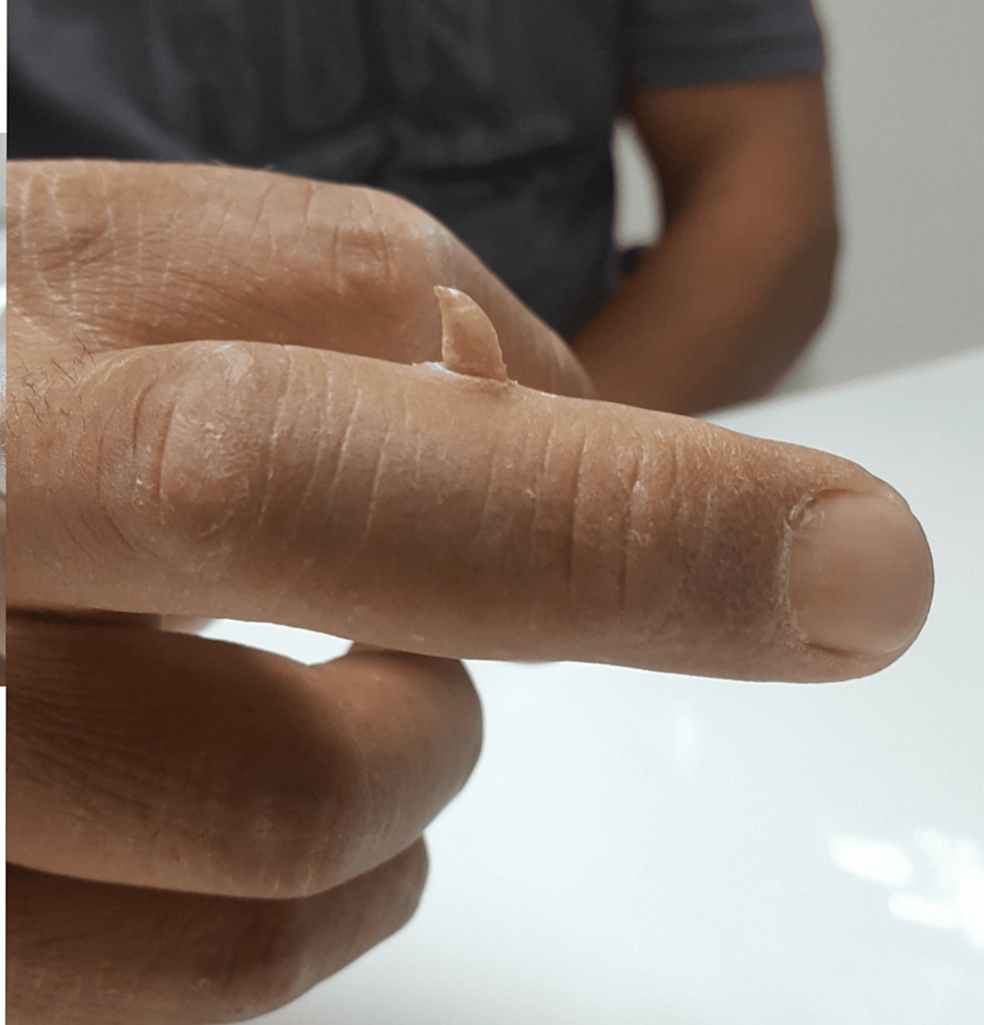
Clinical photograph Upon examination, the lesion presented like a 1 cm long cutaneous horn, emerging from a hyperkeratotic collarette.

Upon examination, the lesion presented like a 1 cm long cutaneous horn, emerging from a hyperkeratotic collarette (Figure 1).

**Figure 2:**
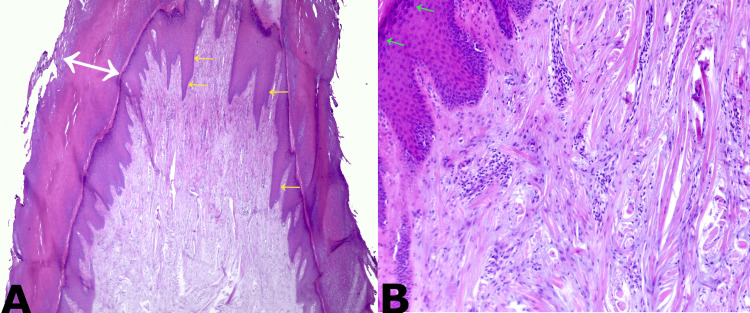
Histopathological photographs (A) H&E 40x. Dermal proliferation composed of dense collagen fibers (oriented predominantly in the vertical direction) and blood vessels covered by keratinizing mature squamous epithelium with papillomatosis (yellow arrows) and marked compact hyperkeratosis (white arrows). Both neural and adnexal structures absent. (B) H&E 200x. Hypergranulosis (green arrows) and thin-walled capillaries and simple endothelium with congestion within dermal papillae. Thickened collagen within the dermis oriented predominantly in the vertical direction.

Excision was performed by shaving and electrocoagulation-curettage of the base. Histological sections stained with hematoxylin and eosin showed a fibroepithelial structure covered by keratinizing mature squamous epithelium with papillomatosis, hypergranulosis, and marked compact hyperkeratosis, with an axis of dense, vascularized connective tissue, without cellular atypia. Both neural and adnexal structures were absent in the lesion. In some dermal papillae, thin-walled capillaries and simple endothelium with congestion were found (Figure 2).

In a three-year follow-up, there was no evidence of recurrence of the removed lesion, nor the appearance of new ones.

## Discussion

The term fibrokeratoma was initially used by Paul Gerson Unna in 1896. It denotes a fibroepithelial tumor with a fibrous center and a hyperkeratotic epithelium, usually found on the eyelids or neck of older adults. These tumors were later interpreted by histopathologists as acrochordons or warts [3].

The term acquired digital fibrokeratoma was first used in 1968 by Bart et al. to describe benign acquired lesions commonly located on the fingers that appear as firm, more or less hyperkeratotic projections arising from a collar of raised skin and resembling supernumerary rudimentary fingers [4]. However, these lesions can also occur on the palms, soles, and toes, which is why the names “acquired fibrokeratoma” and “acral fibrokeratoma” were suggested. Depending on their location, they may also be called “periungual acquired fibrokeratoma” or “subungual acquired fibrokeratoma” [5]. Although the term “digital” is used to describe ADFK, there have also been cases described in the literature of ADFK-type tumors in the heel, back of the hands, back of the wrists, ankles, and prepatellar region [2].

ADFK has been reported in people of all races [6], more commonly in adult men in the age range of 39 to 77 years [3]. It has been hypothesized that ADFK occurs as a result of trauma to the affected region, either via a single major event or multiple minor episodes [2]. However, the antecedent is only in a few cases. Some studies suggest the participation of factor XIIIa in the pathogenesis of ADFK [5].

Clinically, the lesion presents as a solitary, asymptomatic, flesh-colored, dome-shaped papule, although it can also be elongated or pedunculated, which is usually less than 1 cm long, mainly on the fingers or toes [1,5]. Exceptionally, cases of giant acquired digital fibrokeratomas have been reported [7].

In 1985, Kint et al. reported three different variants based on histopathologic and clinical features. Type l is a domed-shaped lesion that has a core of thick, densely packed collagen bundles with fine elastic fibers and a hyperkeratotic epidermis. Type ll is a mainly tall and hyperkeratotic lesion that contains more fibroblasts in the cutis and is more regularly arranged than the first type with reduced elastic fibers. Type III is a flat to dome-shaped lesion with a poorly cellular structure and absent elastic fibers. All three types share a very hyperkeratotic and acanthotic epidermis [8].

Differential diagnoses include common warts, which can be easily identified by their dermoscopic characteristics such as tiny black dots. Superficial acral fibromyxoma, which can be differentiated by its histopathological characteristics on H&E stain. Supernumerary digit, which can be suspected when the lesion is present since birth, and can also be differentiated by its histopathological characteristics on H&E stain. Periungual fibroma (or Koenen's tumor) is classically associated with tuberous sclerosis; these types of tumors can be differentiated by their histopathological characteristics. Nonpigmented eccrine poroma can be differentiated by its histopathological characteristics on both their pigmented and non-pigmented variants: Pyogenic granuloma, which has a characteristic history of rapid growth rate, and a friable surface that can easily bleed with trauma, also has several dermoscopic characteristics. Keratoacanthoma has dermoscopic differences and histopathological characteristics not found in ADFKs. Cutaneous neurofibroma requires histopathological examination, usually with immunohistochemical stains, to diagnose. Enchondroma can be differentiated by its content's consistency, and by histopathological examination. Dermatofibroma has several dermoscopic characteristics and can be differentiated by histopathological examination. Aggressive digital papillary adenocarcinoma can only be differentiated by histopathological examination with immunohistochemical stains [5].

To date, there have been no reported cases of spontaneous remission of ADFKs, but surgical excision is curative and recurrence is rare [5].

## Conclusions

The case we present coincides both clinically and histopathologically with the first descriptions of acquired digital fibrokeratoma. However, due to the varied locations reported, not always acral, we believe the term "acquired fibrokeratoma" to be more accurate to describe this type of tumor in other locations.

We present this case to highlight the patient's history, who had a clear history of multiple traumas, and an unusual clinical course, with spontaneous remissions followed by new growth of the tumor. Both spontaneous remission and spontaneous recurrence have not been previously described in the literature.
